# Tracking Single Cells Motility on Different Substrates

**DOI:** 10.3390/mps3030056

**Published:** 2020-08-04

**Authors:** Pooja Sharma, Van K. Lam, Christopher B. Raub, Byung Min Chung

**Affiliations:** 1 Department of Biology, The Catholic University of America, Washington, DC 20064, USA; 48sharma@cua.edu; 2 Department of Biomedical Engineering, The Catholic University of America, Washington, DC 20064, USA; 75lam@cua.edu

**Keywords:** substrate stiffness, collagen extracellular matrix, single cell motility, live cell tracking, wrMTrck plugin, ImageJ

## Abstract

Motility is a key property of a cell, required for several physiological processes, including embryonic development, axon guidance, tissue regeneration, gastrulation, immune response, and cancer metastasis. Therefore, the ability to examine cell motility, especially at a single cell level, is important for understanding various biological processes. Several different assays are currently available to examine cell motility. However, studying cell motility at a single cell level can be costly and/or challenging. Here, we describe a method of tracking random cell motility on different substrates such as glass, tissue-culture polystyrene, and type I collagen hydrogels, which can be modified to generate different collagen network microstructures. In this study we tracked MDA-MB-231 breast cancer cells using The CytoSMART^TM^ System (Lonza Group, Basel, Switzerland) for live cell imaging and assessed the average cell migration speed using ImageJ and wrMTrck plugin. Our cost-effective and easy-to-use method allows studying cell motility at a single cell level on different substrates with varying degrees of stiffness and varied compositions. This procedure can be successfully performed in a highly accessible manner with a simple setup.

## 1. Introduction

Both cell motility and migration play crucial roles in tissue homeostasis, regulating several physiological processes, including embryonic development, wound healing, and immune responses [1,2]. In addition, cell motility and migration also serve key functions in cancer during metastasis, as cancer cells migrate and invade into adjacent tissues and blood or lymphatic vessels as they intravasate into blood vessels [3,4]. However, with different modes of cell migration and multiple factors involved [5,6], cell migration is a complex process that is still in need of better understanding.

Cells sense their physical environment and interact dynamically with the extracellular matrix (ECM) as they carry out their functions [7]. Conditions cells encounter may range in compliance from tens of pascals in the softest tissues, such as brain, to gigapascals in the stiffest tissues, such as bone [8,9]. Several studies have reported that optimal stiffness is required for maximum migration potential of cells [8,10,11,12]. Migration of sparsely seeded cells are influenced by matrix rigidity, and cells migrate from a soft to a rigid surface, a mechano-responsiveness referred to as durotaxis [13,14]. At the molecular level, gene expression involved in cell motility was also found to be dependent on the microenvironment and varying substrate stiffness [15,16,17].

Collagen amount and its network microstructure regulates cell migration [18,19]. Collagen is a fibrous protein that is also the most abundant protein in the ECM [20]. In cancer, collagen concentration and fiber structure are suggested to affect tumor malignancy, as cancerous tissue contains 9–45 mg/mL (0.9–4.5% wt.) collagen in the interstitium, while paired normal tissues contain significantly less [21]. As the main structural element of the ECM, collagen provides tensile strength, regulates cell adhesion, supports chemotaxis and migration, and directs tissue development [22]. Studies have revealed that collagen fiber alignment allowed cells to migrate more efficiently by increasing directional persistence [18,23], and collagen was also shown to promote cancer metastasis by activating STAT3 signaling [19].

Collagen matrices can be reconstituted in vitro to exhibit mechanical features that resemble fibrous tissues in vivo and provide a tissue-like environment to study cell behaviors [24]. Among at least 16 types of collagen, type I is by far the most prevalent form and is popular for tissue engineering studies due to its ease of extraction and adaptability for multiple applications [25,26]. Collagen fibrils self-assemble at neutral pH into bundled fibers typically 12–120 nm diameter that crosslink to produce a matrix structure that ultimately forms a hydrogel in the presence of a water-based solvent [27,28].

**Figure 1:**
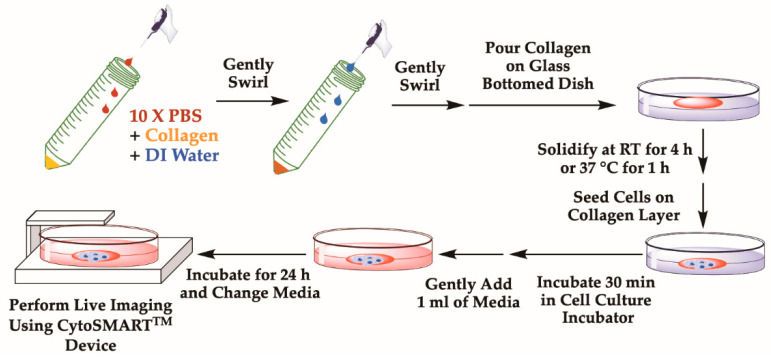
Schematic representation of collagen plate preparation and cell seeding. This process is mainly divided into three parts: (i) collagen solution preparation by diluting collagen with water and adjusting pH with NaOH, poring prepared collagen solution on to glass bottom plates; (ii) cell seeding on collagen layer and incubating for 30 min for cells to attach on collagen surface; (iii) next day, replacing old media with fresh media and then performing live cell imaging using the CytoSMART^TM^ System.

Various in vitro and in vivo cell migration assays have been developed over the years, including the Boyden chamber assay [29], scratch assay/wound healing assay [30], three-dimensional (3D) collagen matrix migration assay [31], time-lapse movies, tracking the migration of cultured or tissue-embedded cells [32,33], matrix-permeation assays [29,34], recording of the cell migration based on assays such as phagokinetic tracks formation [35,36], as well as intravital imaging/microscopy of cells in vivo [37]. Each of these cell motility and migration assays has pros and cons, e.g., concerning costs and need of equipment and handling or reliability of obtaining data. However, most of these assays can only examine cells in bulk, are limited in studying cell-substrate interactions, and/or do not monitor migration in real time. To overcome these barriers, we here describe a protocol where single cell tracking can be done on different substrates representing various extracellular environments with time-lapse video microscopy. In addition, we provide detailed instructions to prepare collagen hydrogel polymerized at two different temperatures (room temperature (RT) and 37 °C) and at two different concentrations (2 and 4 mg/mL) to assess cell migration dependent on collagen network microstructure and mechanics (Figure 1). Varying the polymerization temperature of type I collagen during hydrogel self-assembly produces a network with larger pores and larger diameter fibers at lower temperatures, with the opposite effect at high temperatures [38]. We also describe a method to track random cell migration using wrMTrck [39] plugin in ImageJ [40]. This technique can easily be used to study cell motility at a single cell level on different substrates such as glass and collagen hydrogels [41]. A protocol using MDA-MB-231 cells is described here but various adherent cell types can be analyzed using this technique.

## 2. Experimental Design for Single Cell Motility Assay

Motility of cells can be assessed on different substrates. Based on the need, cells can be plated on glass, tissue-culture polystyrene or another extracellular matrix substrate such as type I collagen hydrogel (Figure 1). Collagen hydrogels prepared at 2 or 4 mg/mL were polymerized at RT and 37 °C, respectively. Higher collagen concentration and higher polymerization temperature produces a denser fiber density network constituted with thinner fibers and smaller pore-size diameters.

### 2.1. Materials

- Type I collagen from rat tail tendon (Corning, Inc., Corning, NY, USA; Cat# 354249)
- 10× phosphate buffered saline (PBS) (MilliporeSigma, Burlington, MA, USA; Cat# 70011044)
- Trypsin-EDTA (Corning, Inc.; Cat# 25-052-CI)
- Dulbecco’s Modified Essential Medium (GE Healthcare, Logan, UT, USA; Cat# SH30021.01)
- 100 units/mL penicillin-100 μg/mL streptomycin (Caisson lab, Smithfield, UT, USA; Cat# PSL01-100ML)
- Fetalgro bovine growth serum (RMBIO, Missoula, MT, USA; Cat# FGR-BBT)
- 1M sodium hydroxide (NaOH) (Fisher Scientific, Waltham, MA, USA; Cat# S318-100)
- Parafilm (Southern labware, Cumming, GA, USA; Cat# HS234526A)
- 35 mm diameter glass-bottomed dish (Cellvis, Mountain View, CA, USA; Cat# D35-20-0-N)
- De-ionized (DI) water

### 2.2. Equipment and Software

- Cell culture incubator
- Laminar air flow
- Centrifuge
- Hemocytometer
- ImageJ software (NIH, Bethesda, MD, USA)
- 250 µm transmission electron microscopy copper grid (TED Pella, Inc., Redding, CA, USA)
- Olympus CK2 Inverted Trinocular Phase Tissue Culture Microscope (OLYMPUS OPTICAL CO. LTD., Japan) equipped with an AM Scope 3.7 digital camera (AmScope, Irvine, CA, USA)
- CytoSMART^®^ device (Lonza Group, Basel, Switzerland)

Note: “CytoSMART Lux2” is the latest version of CytoSMART^®^ 2 device with similar features and operating procedure.

## 3. Procedure

- **Collagen Preparation: Time for Completion: 00:30 h**
  Note: All reagent used should be sterile and the work should be performed under the sterile hood; collagen preparation should be done on ice.
  1. To prepare collagen solution of a final concentration at X mg/mL, add the required volume of type I collagen into a sterile 50 mL falcon tube.
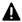
 **CRITICAL STEP** Because of the viscosity of collagen solution, be slow when adding collagen.
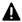
 **CRITICAL STEP** Use a 50 mL tube so that collagen can be mixed easily with gentle swirls in subsequent steps.
  2. Collagen should also be mixed with 1 part of sterile 10× PBS supplemented with phenol red, which is used to indicate the pH of collagen solution (Figure 1).
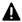
 **CRITICAL STEP** While making collagen solution, avoid air bubble formation by gently pipetting collagen solution, as bubbles cannot be removed easily once introduced into collagen solution.
  3. The final volume should be adjusted with sterile DI water.
    o For instance, if one is making 500 μL of 4 mg/mL of collagen, take out 250 μL of collagen solution (if stock is 8 mg/mL), add 50 μL of 10X PBS, add 150 μL of water to make a final volume of 500 µL.
  4. If the pH of the collagen solution is acidic, the color of the solution will be yellow.
    o If the solution is yellow, carefully add 0.1 M NaOH dropwise and examine the color of the solution. As NaOH is added, the color of the solution will shift from yellow to orange-red, which indicates that the pH is around 7.4–7.6 (Figure 1).
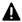
 **CRITICAL STEP** The mixture should be kept cold while adjusting the pH.
  5. Mix the entire collagen solution by gently swirling the tube and keep the tube in an ice bucket until you are ready to pour the mixture into a plate.
  6. Add the final collagen solution onto a glass surface of a 35 mm diameter glass-bottomed dish (Figure 1).
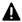
 **CRITICAL STEP** While adding collagen, avoid introducing bubbles on the dish. Swirl the dish to uniformly distribute collagen over the surface of the glass-bottomed area. In total, 250 μL of collagen solution is required to fully cover the glass bottom surface of a dish 35 mm in diameter.
  7. Wrap the collagen-coated dish with parafilm to avoid evaporation and pH change. The dish can be left for 1 h at 37 °C or 4 h at RT to obtain different structures of collagen network.
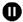
 **PAUSE STEP** The dish can instead be left overnight at 4 °C.
  8. **OPTIONAL STEP** Collagen concentration and polymerization condition described here have been optimized for MDA-MB-231 cells. Therefore, substrate concentration or polymerizing condition can be modified for different cells or substrate types.
- **Cell Seeding (Steps #1 to 6 can vary based on cell type): Time for Completion: 01:00 h**
  1. Wash adherent cancer cells growing on a 10 cm tissue culture-treated dish with 5 mL of 1X PBS. Remove PBS.
  2. Add 1 mL of 0.05% trypsin-EDTA to each dish and return cells to the tissue culture incubator (37 °C and 5% CO2). Incubate for 5 min or until cells are detached from the bottom of the dish.
  3. Quench trypsin by adding trypsin inhibitor or >4X the volume (4 mL) of cell culture medium.
  4. Vigorously pipette the 5 mL solution up and down to create single cell suspension.
  5. Transfer 5 mL of single cell suspension to a 15 mL conical tube and count number of cells using a hemocytometer or cell counter.
  6. Centrifuge the solution at 1000× *g* for 3 min. Aspirate the solution and resuspend the cell pellet in fresh cell culture medium so that cell number is 1 million cells/mL.
  7. Make 200 μL of cell suspension using complete growth medium.
    o For MDA-MB-231 cells, use 30,000 to 35,000 cells in Dulbecco’s modified essential medium DMEM containing 10% Fetalgro bovine growth serum, 100 units/mL penicillin, and 100 μg/mL streptomycin.
    o For 30,000 cells, add 30 uL of suspended cells in 170 μL of complete medium. Mix them well.
  8. Carefully add cell suspension dropwise on the glass surface or collagen layer (Figure 1).
    o Gently swirl the dish to distribute cells evenly as cells are being dropped.
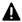
 **CRITICAL STEP** If plating cells on a collagen coated dish, be careful not to damage the existing collagen layer while plating cells.
  9. Place the dish inside the tissue culture incubator for 30–40 min to allow cells to attach to the substrate.
  10. After the incubation, add 1.5 mL of complete growth medium to the plate and incubate for 24 h in the tissue culture incubator to allow cells to completely attach to their substrate.
  11. The next day, gently remove the old media.
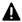
 **CRITICAL STEP** It is best to use a pipette, rather than using an aspirator, as the latter can remove the cell/substrate layer as well.
  12. Add 3 mL of fresh growth medium to the plate.
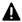
 **CRITICAL STEP** Do so gently so that cell/collagen mixture remains undisturbed.
- **Setting Up Live Cell Imaging:**

The CytoSMART^TM^ System is a live cell imaging system by Lonza and monitors cells without disturbing the cultures during incubation. Since the system is integrated with cloud functionality, the CytoSMART device allows users to monitor cells remotely in real-time using any browser-capable devices which includes computers, smartphones, and tablets.

3A. Setting up the CytoSMART^TM^ System for Live Imaging: Time for Completion: 24:15 h
  1. Disinfect the device by 70% Ethanol and place the CytoSMART^TM^ device inside the tissue culture incubator.
  2. Run the CytoSMART^TM^ cable between the door and the frame of the incubator.
  3. The cable connecting the CytoSMART^TM^ device and a monitoring tablet should be well connected.
  4. Open the incubator and check to see if the light panel around the CytoSMART^TM^ device is illuminated.
    o Blue light: no project running; Red light: project running.
  5. Turn on the tablet connected to the CytoSMART^TM^ device.
  6. Place a dish containing cells on the CytoSMART^TM^ device.
  7. Observe cells on the tablet screen. Adjust the focus and brightness of cells using the slider to generate good contrast between cells and the background.
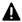
 **CRITICAL STEP** The plus and minus buttons can be used for more precise adjustments.
  8. To start recording, select “Project”.
  9. Select the “Project Name” and use the on-screen keyboard to give the project a name.
    **OPTIONAL STEP** Extra notes can be added if desired (e.g., cell type, passage, etc.).
  10. Select “Next” and enter your email address to receive the project link.
  11. Select “Next” and select the desired recording frequency.
  12. Use the drop-down menu to select an interval of 5 min.
  13. Select the “Start” button. The system will immediately enter sleep mode. Tap anywhere on the screen to re-activate the screen.
    o An email will be sent with a link to open the project.
  14. After 24 h of recording, select the red “Stop project” button to finish the project.
    o The system will send an email indicating that the project has ended.
  15. To access the project, select the “View” button in the email. This will take the user to CytoSMART^TM^ Connect Project Page where cells can be monitored remotely with any browser-capable device. Once the project is finished, the data can be downloaded as a movie in avi format and series of images.
  16. Downloaded data will be used for cell tracking analysis.
3B. Motility Analysis

General Considerations

**Figure 2:**
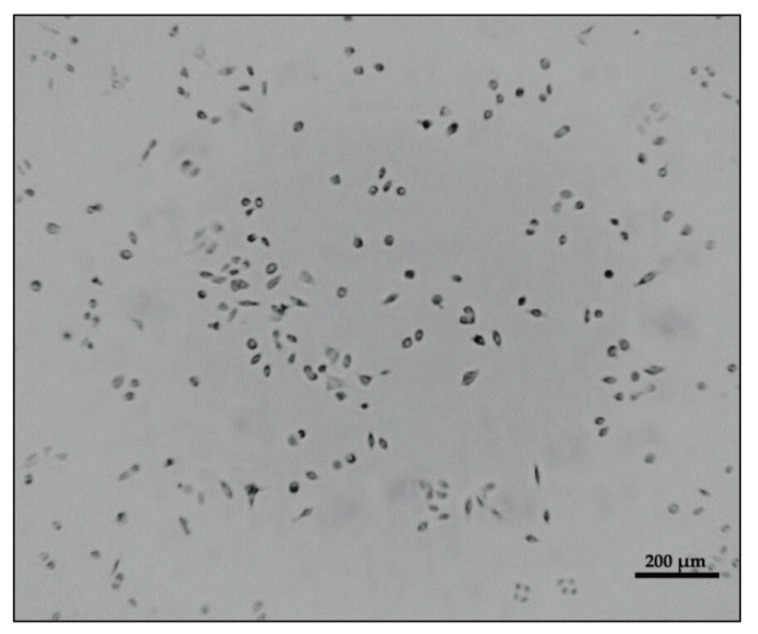
An image of cells using the CytoSMART^TM^ device. Cells appear as dark objects against a brighter background.

In order for this software to locate and track motion of cells, the use of reasonably quality images are required. Images of reasonable quality are those that contain proper contrast without being too bright and ones where cells appear dark (if possible, with no highlights) on a brighter background (Figure 2).

**Figure 3:**
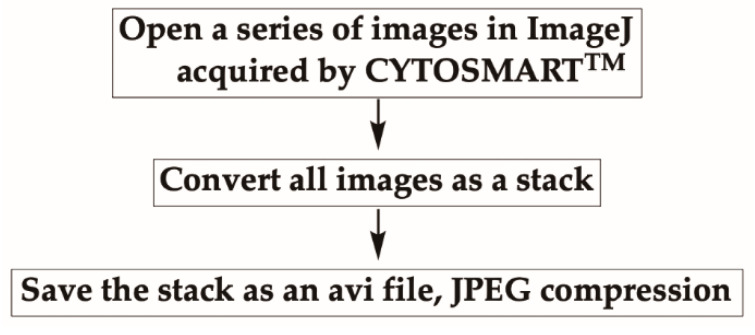
Converting images as an avi file in ImageJ. All images captured at 5 min intervals using the CYTOSMART^TM^ device are opened and converted to a stack. The converted stack is saved as an avi file, in JPEG compression with 7 frames per second (fps).

**Figure 4:**
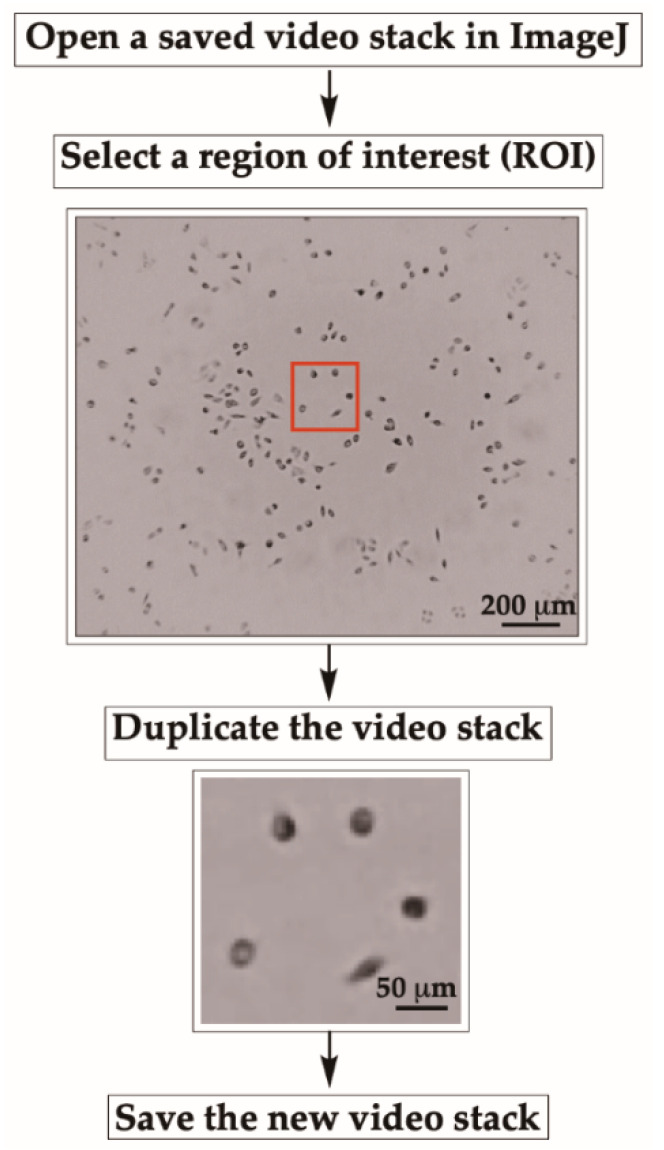
Tracking cells using wrMTrck. Region of interest (ROI) is cropped for cell tracking using rectangular selection tool in ImageJ. The ROI is duplicated and saved as a new stack in avi format.

**Figure 5:**
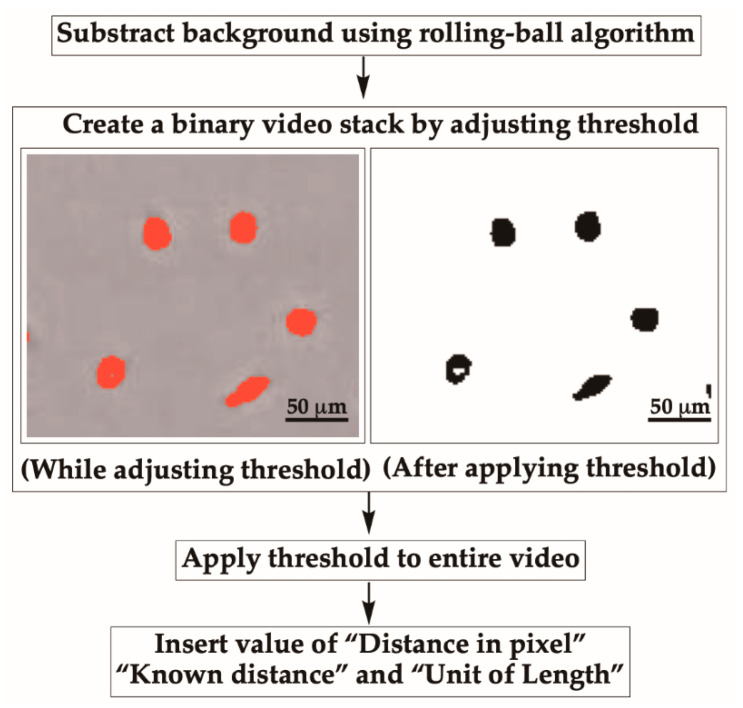
Thresholding the movie and converting to binary using ImageJ. Adjust the threshold so that cells become red, limiting the appearance of red spots in the background. Further set the scale by inserting the value of “Distance in pixels,” “Known distance,” and “Unit of length.”

3B-1. Convert Images to Stack Using ImageJ
  1. Open a series of images in a folder acquired by the CYTOSMART^TM^ system in ImageJ, by either dragging the folder containing images onto the ImageJ bar or by selecting option “File” then “Open” from the top menu.
  2. Once all images are opened, convert them as a stack by selecting “Images” then “Images to Stack” from the menu.
  3. Save the stack as an avi file, JPEG compression with 7 fps (frames per second) (Figure 3).
3B-2. Select the Frame to Analyze
  As cells are grown for 24 h, there may be lot of debris or cells in clusters appearing in a recorded frame, which can interfere with the analysis. To avoid this problem, choosing a field of view containing no more than 10 isolated cells is preferred.
  1. To start cell motility analysis, open a video stack (from 3B-1) in ImageJ.
  2. Select a region of interest (ROI) with a “specific size of area” by using the rectangular selection tool located at the top left corner of the ImageJ tool bar.
  3. After selecting an area (shown in red box in Figure 4), duplicate the video stack by selecting “Image” then “Duplicate” then “OK” (Figure 4).
  4. Save the new video stack (as described earlier in 3B-1) for further analyses.
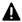
 **CRITICAL STEP** Since the entire field cannot be analyzed at once, a specific region of interest with a sufficient number of cells is cropped, duplicated, and saved as a new stack for further analysis.
  5. Use the same procedure described earlier to select other ROIs with the same size to analyze the motility of all cells in the entire frame.
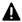
 **CRITICAL STEP** MDA-MB-231 cell area usually changes dramatically over a period of 24 h. They may be more elongated during migration but become rounded during cell division, senescence, or apoptosis. Because of the drastic changes in cell shape, wrMTrck may lose track of a cell during duration of the video. To better trace cells, users should trace few cells at a time; five to 10 cells per ROI is recommended. If necessary, one cell per frame can also be analyzed.
    **OPTIONAL STEP** In some cases where cells leave the frame, reducing the number of stacks can retain the cell movement within the frame.
3B-3. Background Subtraction Using Rolling-Ball Algorithm
  Background subtraction is necessary to every image analysis process, as it helps to correct unevenly illuminated backgrounds caused by uncorrelated fluctuations (Figure S1A).
  1. To remove uneven backgrounds, the rolling-ball method [42], which can remove large spatial variations of the background densities, is applied by selecting the “Process” tab, then choosing “Subtract Background”.
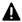
 **CRITICAL STEP** A dialog box will appear asking users to adjust the rolling-ball radius, which should be set to at least the size of the largest object that is not part of the background.
    **OPTIONAL STEP** A preview option can be used to check the result before the actual process.
3B-4. Create Binary Stack
  After the background subtraction, open a video stack. While opening, select “Convert to Grayscale.” Then, create a binary video stack by selecting “Image” and choosing “Adjust” and “Threshold” (Figure 5). Adjust the sliders to find a proper level where only cells become red, limiting the appearance of red spots in the background (Figure 5).
  1. Once an appropriate level is achieved, check the “Stack histogram” box to apply the threshold to the entire video and click “Apply.”
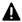
 **CRITICAL STEP** During migration, if cells come close to each other, the wrMTrck algorithm may falsely recognize two cells as one cell. Thus, cells to be analyzed must be visually inspected before running wrMTrck. If cells happen to touch each other, simply separate cells by drawing a line between them using a 1 pixel “Straight line” tool (Figure S1B).
  2. The thresholded movie before and after adjusting the threshold will look like the stack on the right in Figure 5.
  3. Further noise correction to eliminate dots or fill holes in objects can be done by selecting “Process.” Next, either choose “Noise” then “Despeckle” or “Binary” then “Fill Holes.”
  4. Enter in the real-world scale of the cells in the movie by selecting “Analyze” then “Set Scale.”
  5. Insert information for “Distance in pixels,” “Known distance,” and “Unit of length” while maintaining “Pixel aspect ratio” at 1.0. Then click “OK.” If “Distance in pixel” is 164 pixels, “Known distance” is 250 and “Unit of length” is micron; the scale for the analysis would correspond to 0.656 pixels per one micron.

**Figure 6:**
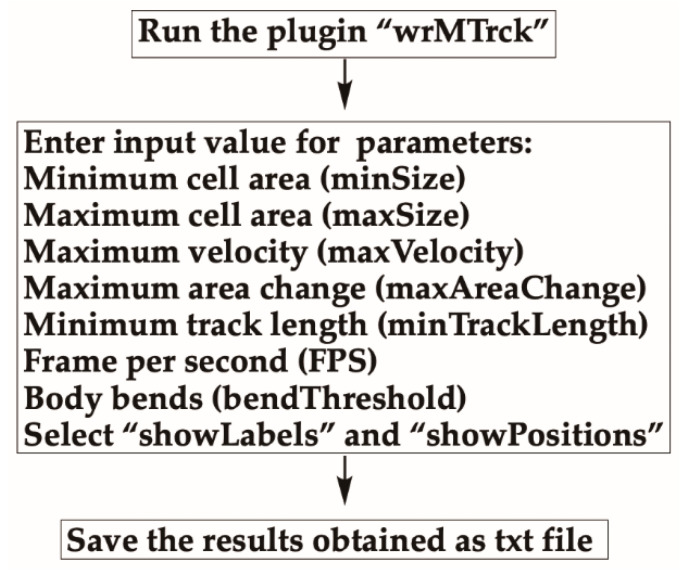
Tracking cells using the wrMTrck plugin. Run the wrMTrck plugin, then determine the average speed of the detected cells by entering in parameters for minSize, maxSize, maxVelocity, maxAreaChange, and minTrackLength. Data value such as average speed and length of tracking will become available in a pop-up window, which can be saved as a txt file.

IV. **Tracking of Cells**
  To track cells, download the wrMTrck plugin (https://www.mrc-lmb.cam.ac.uk/wormtracker/) [39] and install in ImageJ.
  1. Run the wrMTrck plugin by selecting “Plugins” then “wrMTrck” (Figure 6). A dialog box will appear.
  2. Enter values for:
    o The minimum (minSize) and maximum (maxSize) cell area in pixels.
    o maxVelocity: the number of pixels a cell is allowed to travel between two frames.
    o maxAreaChange: the % change in area between consecutive frames allowed—for example, if two cells collide on the plate, each track is broken due to the doubled area.
    o Tracks shorter than “minTrackLength” will be discarded.
    o fps stands for frames per second and is used to calculate the absolute speed of cells per second.
    o bendThreshold is used for detection of body-bends.
    o binSize for speed histogram.
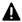
 **CRITICAL STEP** Input parameters should be modified depending on your experiment and cell type analyzed.
  3. Select showLabels to see a final movie where each cell is indicated by a number.
  4. A “Results” window will pop-up containing analyses of cells. Descriptions of column labels are described in Table 1.
  5. Data can be saved as a txt file or can be pasted to an excel sheet for further analyses.

## 4. Results and Discussion

The procedure can be performed on various cell types for time lapse images acquired on various substrate types, and it does not need any further adjustments in most cases. In this report, we monitored the motility of MDA-MB-231 cells on 4 mg/mL collagen polymerized at 37 °C. We selected one random ROI and created new stacks to analyze cell motility over 125 min (Video S1). Input values for MDA-MB-231 cells used were: MinSize: 50, MaxSize: 400, MaxVelocity: 100, MaxAreaChange: 50, and minTrackLength: 30. For other parameters, default values were used.

**Figure 7:**
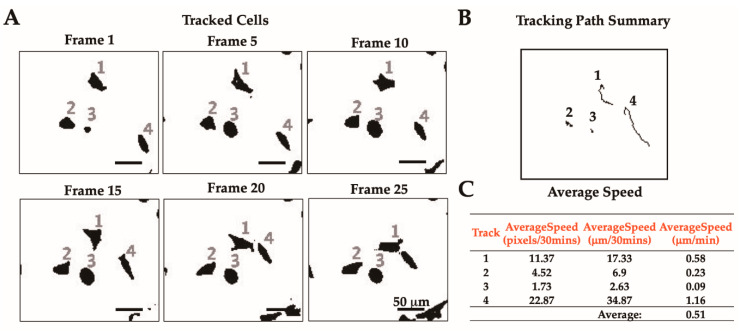
Tracked cells and path summary of MDA-MB-231 cells on 4 mg/mL collagen polymerized at 37 °C. (**A**) Select frames of tracked cells obtained using the wrMTrck plugin. Tracked cells are shown with discrete numbers. (**B**) Summarized tracked paths of all detected cells in multiple frames shown in (**A**). (**C**) The average speed of each tracked cell in pixels/30 min was converted to μm/min.

Results acquired using the wrMTrck plugin consist of tracked cells labelled with discrete numbers (Figure 7A) and a cumulative tracking path summary (Figure 7B). Selected frames from a total of 25 frames representing different time points are presented. A complete report of tracking analyses of cells obtained is reported in Table S1. From the total of 25 frames analyzed, four cells were detected by the wrMTrck plugin. Cells were tracked based on input parameters and cells that were not within the input ranges were excluded. For instance, in frame 1–20, cells #1, #2, #3, and #4 were being tracked whereas in frame 25, only cells #1, #2, and #3 were tracked. Therefore, cells tracked in earlier frames can stop being tracked in later frames. The reason for this could be due to cell #4 being too closely attached to cell #1. Cell tracking can also be terminated by the wrMTrck plugin if cell tracks are shorter than the “minTrackLength” or if cells moved out of the frame. In other instances, wrMTrck will start tracking cells only in later frames when cells meet the criterions in terms of shape and size. At the end, the wrMTrck plugin provides a cumulative tracking path summary for all cells that met the input parameters (Figure 7B).

The tabular form of the result details the quantitated data of different parameters (Table S1). The average speed obtained using the wrMTrck plugin was calculated from Length/Time(s). During the conversion step, the input stack containing images taken by every 5 min was recoded to a video stack with a speed rate of 7 fps. Thus, the average speed unit reported from wrMTrack in our data was designated in pixel/30 min. To determine resolution, a 250 µm transmission electron microscopy copper grid (TED Pella, Inc.) was imaged by CytoSMART^TM^. In ImageJ, 250 µm of the grid’s side bar was measured as 164 pixels. Thus, CytoSMART^TM^ resolution was 1.5243 µm/pixel. Original average speed in pixels/30 min was converted to µm/30 min using the above resolution and then divided by 30 to obtain the final average speed in µm/min (Figure 7C and Table S1). As evident by the obtained speed of the individual cells, the motility of cell #4 was highest (1046.01 μm/min) with the longest path length. The obtained speed from different cells were averaged to get the overall average speed. As a result, MDA-MB-231 cells were found to be migrating at an average speed of 0.51 μm/min on collagen (Figure 7C and Table S1). This result is based on one ROI analysis. To perform a more robust analysis, the number of stacks should be at least three ROIs. Then, the overall average speed can be calculated by averaging the average speed obtained from each stack.

It is crucial that parameters listed in Figure 6 be adjusted for different cells and conditions to best track cells. Inevitably, cells of different types will exhibit sizes and speeds differently from MDA-MB-231 cells. Therefore, proper cutoff parameters should first be established. Analyzing images acquired using an area of a different size also requires adjustments made to input parameters. In addition, cell tracks should be manually confirmed as improper tracking of cells may occur. For example, a cell label may jump from one cell to another, two cells may appear as one cell, or one cell may appear as two cells. As mentioned above, cells can be separated by drawing a line between them using a 1 pixel “Straight line” tool. However, this approach of separating cells manually is time-consuming and will likely make the perimeter measurements incorrect for that frame because of pixel spatial averaging. Still, it has less effect on the cell geometric centroid, and it also forces the user to conduct a quality control check in order to catch and correct such double merged cells. In the future, the ability to examine individual cell motility within a group of physically attached cells will expand the usage of this application.

Though there are many commercial tools available for tracking, ImageJ is one of the most popular imaging software, providing free access allow users to trace and compute dynamic parameters of target objects. Tracking tools of ImageJ can be categorized as either particle or cell tracking based on their purpose. Particle tracking is similar to cell tracking as both techniques requires noise reduction and deconvolution to distinguish objects as bright regions against dark background; however, cell tracking requires a sophisticated analysis of morphology at every time point since cells shape usually changes due to cell growth, division, and apoptosis, which are not included in other popular particle tracking tools such as MTrackJ [43] and MTrack2 [43]. In fact, the wrMTrack plugin was initially established on MTrack2 with advanced functionality allowing users to track cells with dynamic changes in shape by setting a value of maxAreaChange. Using advanced functions such as processing the entire movie with background subtraction, wrMTrack is also able to break tracks once objects exceed size limitation and compute speeds automatically. The automatized cell tracing method used by wrMTrck virtually eliminates manual tracing of cells from frame to frame, which increases both accuracy and time-efficiency of cell trajectory reconstruction. Table S2 summarizes the available ImageJ tracking tools in terms of their target objects, imaging techniques, and applications.

In conclusion, the method described is a simple and cost-effective procedure to track cell motility. It has the advantage of being versatile, since this procedure can be employed to different cells with only minor modifications. Furthermore, this approach allows for a clear interpretation of two-dimensional (2D) migration data as a function of substrate and cell type, including gene knockouts. It can be seen as a first step in the process of assigning substrate-dependence and molecular mechanisms to altered phenotypes. Moreover, movement characteristics of individual cells and groups of cells can be linked to individual and group geometrical parameters. In addition, programs used in this procedure, ImageJ and wrMTrck, are freely accessible and the tracking method can be used from time lapse images acquired using different optical systems. Therefore, this protocol can be used in a wide range of projects including an educational setting, allowing students to easily learn cell behaviors, and can be widely implemented because it uses inexpensive equipment that is widely available.

## Figures and Tables

**Table 1:** Description of all column labels of the tracking analysis.

Column Label	Description
Length	Sum of length of all movement vectors between frames given track [42].
Distance	Distance covered by cell from start to finish.
#Frames	Number of frames the cell was tracked.
1stFrame	The first frame where the cell is tracked.
Time(s)	The time cell was tracked (zero if the fps value is not detected correctly).
MaxSpeed	Maximum distance (pixels) covered by one cell between two frames.
Area	Average area of the cell tracked (in this case in mm^2^).
sdArea	Standard deviation of the area.
Perim	The length the average perimeter (outline) of the cell tracked. This number is approximately two times the length of the cell (i.e., 1.1–1.25 mm in this case). Tracks with abnormal lengths could indicate that two cells moved together as one.
sdPerim	Standard deviation of the perimeter. You may want to discard cells with large sdPerims, since this could indicate a collision between cells that was not detected by the wrMTrck plugin.
AvgSpeed	The average speed calculated from Length/Time(s).
BLPS	Body Lengths Per Second, calculated by dividing the Length of the track by Perim/2 and the time(s).
avgX	The average X-coordinate of the track.
avgY	The average Y-coordinate of the track.
Bends	Counting the number of body-bends for a few of the tracks
BBPS	Counting the body bends per seconds

## Supplementary Materials

The following are available online at https://www.mdpi.com/2409-9279/3/3/56/s1. Figure S1: Background subtraction using a rolling-ball algorithm and a straight-line tool used to separate attached cells. (**A**) Background subtraction using a rolling-ball algorithm helps to correct unevenly illuminated background caused by uncorrelated fluctuations. A representative frame before and after background subtraction are presented. (**B**) Attached cells are separated by drawing a line between them using a 1 pixel “Straight line” tool. Cells in a red box are attached to each other in frame 19 and frame 20. The Straight line tool was used to separate them; Table S1: Data values obtained for cell motility analyses using wrMTrck plugin in ImageJ; Table S2: Comparison and summary of cell tracking tools available in ImageJ; Video S1: Detection and tracking of cells motility using ImageJ.
