# Supplementary material for: Tracking Single Cells Motility on Different Substrates
Source: Methods Protoc. 2020 Aug 4;3(3):56. doi: 10.3390/mps3030056 (PMC7564475; doi:10.3390/mps3030056)
Supplement: Supplementary file 1 [file mps-03-00056-s001.zip › Sharma & Lam et al Supplementary material.pdf]

Supplementary Material

Tracking Single Cells Motility on Different Substrates

Pooja Sharma, Van K. Lam, Christopher B. Raub, and Byung Min Chung

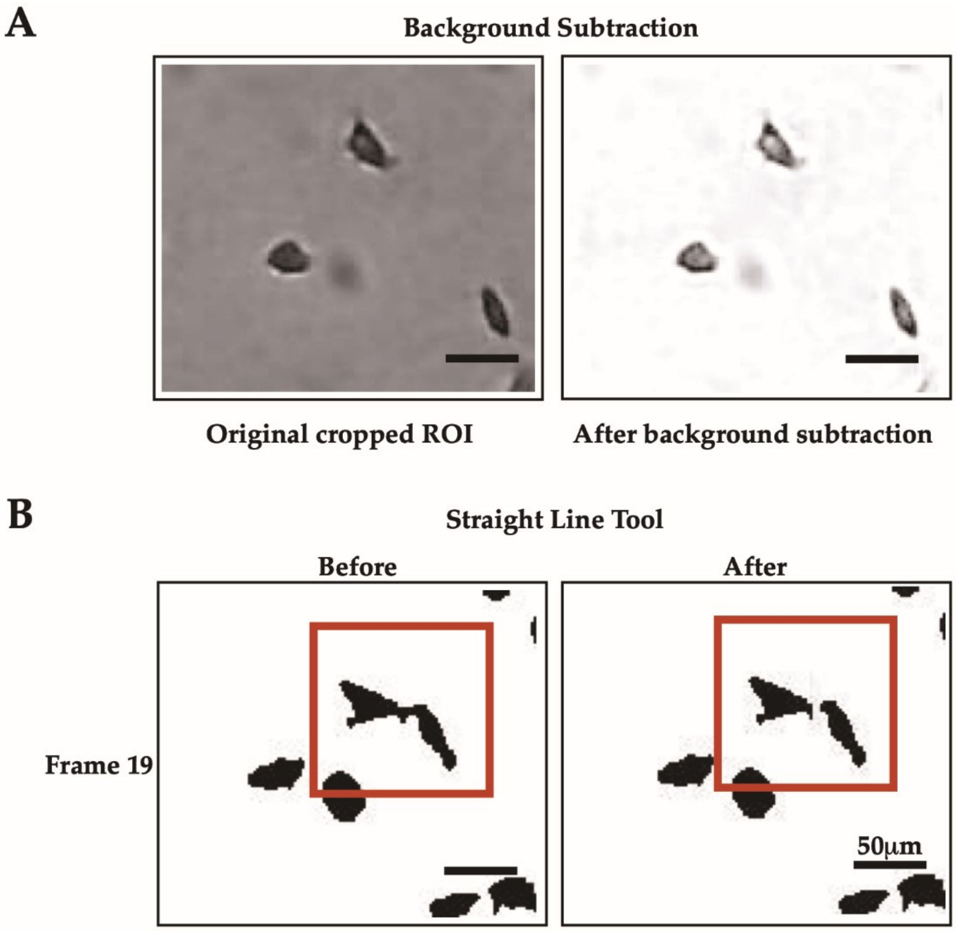

Figure S1 Background subtraction using rolling-ball algorithm and straight-line tool used to separate attached cells. A) Background subtraction using rolling-ball algorithm_helps to correct unevenly illuminated background caused by uncorrelated fluctuations. A representative frame before and after background subtraction are presented. B) Attach cells are separated by drawing a line between them using a 1 pixel “Straight line” tool. Cells in a red box are attached to each other in frame 19 and frame 20. Straight line” tool used to separate them.

Table S1. A complete report of cell tracking from Figure 7

Track	Length	Distance	#Frames	1stFrame	Time(s)	MaxSpeed	Area (in micron)	sdArea	Perim	sdPerim	AverageSpeed (in pixels/30mins)	BLPS	avgX	avgY	Bends	BBPS	AverageSpeed (in pixels/30mins)	AverageSpeed (in micron/30mins) {164 pixels = 250micron}	AverageSpeed (in micron/min)
1	40.603	26.609	25	0	3.571	0	502.119	67.212	115.624	20.844	11.369	0.197	118.731	58.164	0	0	11.369	17.3297667	0.57765889
2	16.156	7.691	25	0	3.571	0	383.514	29.004	82.34	7.656	4.524	0.11	67.088	108.686	0	0	4.524	6.8959332	0.22986444
3	6.169	2.447	25	0	3.571	0	431.291	101.244	76.307	11.711	1.727	0.045	103.215	119	0	0	1.727	2.6324661	0.08774887
4	75.157	60.3	23	0	3.286	0	318.356	37.984	85.938	15.23	22.874	0.532	170.461	113.602	0.5	0.152	22.874	34.8668382	1.16222794
																		Average	0.514375035

Cell	AverageSpeed (in pixels/30mins)	AverageSpeed (in micron/30mins)	AverageSpeed (in micron/min)
1	11.37	17.33	0.58
2	4.52	6.90	0.23
3	1.73	2.63	0.09
4	22.87	34.87	1.16
		Average	0.51

Table S2: A summary of available tracking tools of ImageJ

Name	Object Specific	Microscopy	Resolution Required	Dimension	Automation	Application
LineageTracker [46]	Cell	Fluorescence microscopy	None	2D	Auto	Tracking large displacement of cells from frame to frame
QuimP [47]	Cell	Fluorescence microscopy	Yes	2D	Auto	Tracking changes in cellular shape distribution of fluorescent labels inside cells
MTrackJ [48]	Particle	Phase Bright-field	None	3D	Manual	Tracking moving objects and measuring tracking parameters
MultiTracker [48]	Particle	Phase Bright-field	None	2D	Manual	Tracking object movement
MTrack2[48]	Particle	Phase Bright-field	None	2D	Auto	Identifying objects in each frame based on given parameters, then tracking those objects' movement in the following successive frames
WrmTrack [49]	Worm	Phase Bright-field	None	2D	Auto	Similar to MTrack2 but more specific to living organisms.
